# Nervous necrosis virus capsid protein and Protein A dynamically modulate the fish cGAS-mediated IFN signal pathway to facilitate viral evasion

**DOI:** 10.1128/jvi.00686-24

**Published:** 2024-06-18

**Authors:** Siyou Huang, Linwei Yang, Rui Zheng, Shaoping Weng, Jianguo He, Junfeng Xie

**Affiliations:** 1 State Key Laboratory of Biocontrol, Southern Marine Science and Engineering Guangdong Laboratory (Zhuhai), China-ASEAN Belt and Road Joint Laboratory on Mariculture Technology, Guangdong Provincial Key Laboratory of Aquatic Economic Animals, School of Life Sciences, Sun Yat-sen University, Guangzhou, China; University of Michigan Medical School, Ann Arbor, Michigan, USA

**Keywords:** nervous necrosis virus, cGAS, CP, ProA, UBE3C, innate immune response

## Abstract

Nervous necrosis virus (NNV), an aquatic RNA virus belonging to *Betanodavirus*, infects a variety of marine and freshwater fishes, leading to massive mortality of cultured larvae and juveniles and substantial economic losses. The enzyme cyclic guanosine monophosphate-adenosine monophosphate synthase (cGAS) is widely recognized as a central component in the innate immune response to cytosolic DNA derived from different pathogens. However, little is known about the response of cGAS to aquatic RNA viruses. This study found that *Epinephelus coioides* cGAS (EccGAS) overexpression inhibited NNV replication, whereas EccGAS silencing promoted NNV replication. The anti-NNV activity of EccGAS was involved in interferon (IFN) signaling activation including tumor necrosis factor receptor-associated factor family member-associated NF-kappa-B activator-binding kinase 1 (TBK1) phosphorylation, interferon regulatory factor 3 (IRF3) nuclear translocation, and the subsequent induction of IFNc and ISGs. Interestingly, NNV employed its capsid protein (CP) or Protein A (ProA) to negatively or positively modulate EccGAS-mediated IFN signaling by simultaneously targeting EccGAS. CP interacted with EccGAS via the arm-P, S-P, and SD structural domains and promoted its polyubiquitination with K48 and K63 linkages in an EcUBE3C (the ubiquitin ligase)-dependent manner, ultimately leading to EccGAS degradation. Conversely, ProA bound to EccGAS and inhibited its ubiquitination and degradation. In regulating EccGAS protein content, CP’s inhibitory action was more pronounced than ProA’s protective effect, allowing successful NNV replication. These novel findings suggest that NNV CP and ProA dynamically modulate the EccGAS-mediated IFN signaling pathway to facilitate the immune escape of NNV. Our findings shed light on a novel mechanism of virus-host interaction and provide a theoretical basis for the prevention and control of NNV.

**IMPORTANCE:**

As a well-known DNA sensor, cGAS is a pivotal component in innate anti-viral immunity to anti-DNA viruses. Although there is growing evidence regarding the function of cGAS in the resistance to RNA viruses, the mechanisms by which cGAS participates in RNA virus-induced immune responses in fish and how aquatic viruses evade cGAS-mediated immune surveillance remain elusive. Here, we investigated the detailed mechanism by which EccGAS positively regulates the anti-NNV response. Furthermore, NNV CP and ProA interacted with EccGAS, regulating its protein levels through ubiquitin-proteasome pathways, to dynamically modulate the EccGAS-mediated IFN signaling pathway and facilitate viral evasion. Notably, NNV CP was identified to promote the ubiquitination of EccGAS via ubiquitin ligase EcUBE3C. These findings unveil a novel strategy for aquatic RNA viruses to evade cGAS-mediated innate immunity, enhancing our understanding of virus-host interactions.

## INTRODUCTION

The innate immune response is the first defense against pathogenic infections (1). This response is generally triggered via the detection of evolutionarily conserved pathogen-associated molecular patterns (PAMPs) by a diverse group of host pattern recognition receptors (PRRs) (1). The major PAMPs, viral nucleic acids, are sensed by cellular PRRs after virus infection (2). The enzyme cyclic guanosine monophosphate-adenosine monophosphate synthase (cGAS) is considered the major PRR responsible for sensing viral DNA (3–6), while the retinoic acid-inducible gene I (RIG-I)-like receptors, including RIG-I, melanoma differentiation-associated gene 5 (MDA5), and laboratory of genetics and physiology 2 (LGP2), are recognized as viral RNA sensors (7–10). After sensing the viral DNA, cGAS can induce the activation of downstream effector molecules such as the stimulator of interferon genes (STING) and tumor necrosis factor receptor-associated factor family member-associated NF-kappa-B activator-binding kinase 1 (TBK1) (11, 12). TBK1 then interacts with STING and further phosphorylates interferon regulatory factor 3 (IRF3), which forms a dimer and translocates to the nucleus to activate the expression of type I interferon (IFN-I) (6, 11, 13, 14). IFN-I can trigger the expression of interferon (IFN) pathway-related factors, including interferon-induced protein with tetratricopeptide repeats 1 (IFIT1) (15), interferon-stimulated gene 15 (ISG15) (16), and myxovirus resistance gene 1 (Mx1), to exert anti-viral function (17, 18). In addition, activation of IRF3, together with nuclear factor-kappaB (NF-κB), induces the expression of proinflammatory cytokines such as interleukin (IL)-1β, IL-6, IL-8, and tumor necrosis factor-alpha (TNF-α) (19, 20).

Although cGAS is widely considered to be a DNA sensor, there is evidence that cGAS is also engaged in anti-viral responses to RNA viruses, suggesting a complex cross-talk between the innate perception of cytoplasmic DNA and RNA (21–23). Studies have shown that cGAS knockout mice had higher infection and mortality rates than wild-type mice when infected with West Nile virus (WNV) (24), and the lack of cGAS significantly promoted the replication of several RNA viruses, including Sendai virus (SeV), vesicular stomatitis virus, WNV, and dengue virus (DENV) (24–27). Subsequently, Sun *et al.* demonstrated that mitochondrial DNA was released into the cytoplasm to activate the cGAS during DENV infection (28). In fish, it has been found that cGAS was involved in RNA virus infection (29–31); however, cGAS has shown dual action in the context of aquatic RNA virus infection. For example, cGASa in grass carp *Ctenopharyngodon idellusc* was effective in resisting grass carp reovirus (GCRV) infection, while grass carp cGASb was in favor of GCRV replication (31). Hence, there is a need for further research on the role of cGAS in aquatic RNA viruses.

Nervous necrosis virus (NNV) is an aquatic RNA virus belonging to the genus *Betanodavirus* within the *Nodaviridae* family. The mortality rate of NNV-infected larvae and juvenile fish is up to 100%, which is currently the greatest threat to the grouper aquaculture industry. The NNV genome consists of two single-stranded RNAs (RNA1 and RNA2) that generate subgenome RNA3 during replication. NNV contains three well-defined open reading frames (ORFs) encoding the structural protein capsid protein (CP) and the non-structural proteins Protein A [ProA, aka RNA-dependent RNA polymerase (RdRp)], and B2 (32). There is also a fourth contentious ORF known as B1, which has been reported as absent in Atlantic halibut nodavirus (33), a member of the Barfin flounder NNV genotype, but present in the red-spotted grouper NNV (RGNNV) genotype (34), where it plays as a transcript antagonist to inhibit the IFN response (35). Our recent findings demonstrated that ProA of orange-spotted nervous necrosis virus (OGNNV), a member of the RGNNV genotype, effectively triggers the production of IFN through the RIG/MDA5-MAVS-TBK1-IRF3 signal pathway, resulting in an anti-viral response (36). The CP of NNV interacts with *Lateolabrax japonicus* ring finger protein 34 and subsequently facilitates the degradation of LjIRF3 through ubiquitination, ultimately inhibiting the production of IFN (37). Furthermore, NNV CP has been shown to suppress the production of IFN by facilitating the ubiquitin-mediated degradation of LjTRAF3 via LjRNF114 (38). However, there remains extremely limited information concerning the escape mechanism of NNV, and further research is urgently needed.

The orange-spotted grouper *Epinephelus coioides*, an economically important fish, is susceptible to NNV infection. In this study, we found that cGAS, a well-known DNA recognition receptor, was involved in the resistance to RNA virus OGNNV. The CP and ProA of NNV interacted with *Epinephelus coioides* cGAS (EccGAS) simultaneously to dynamically regulate IFN signaling. Our findings unveil a novel strategy by which NNV regulates innate immunity and evades host anti-viral responses.

## RESULTS

### OGNNV infection induces the expression of EccGAS and EcSTING

**Fig 1:**
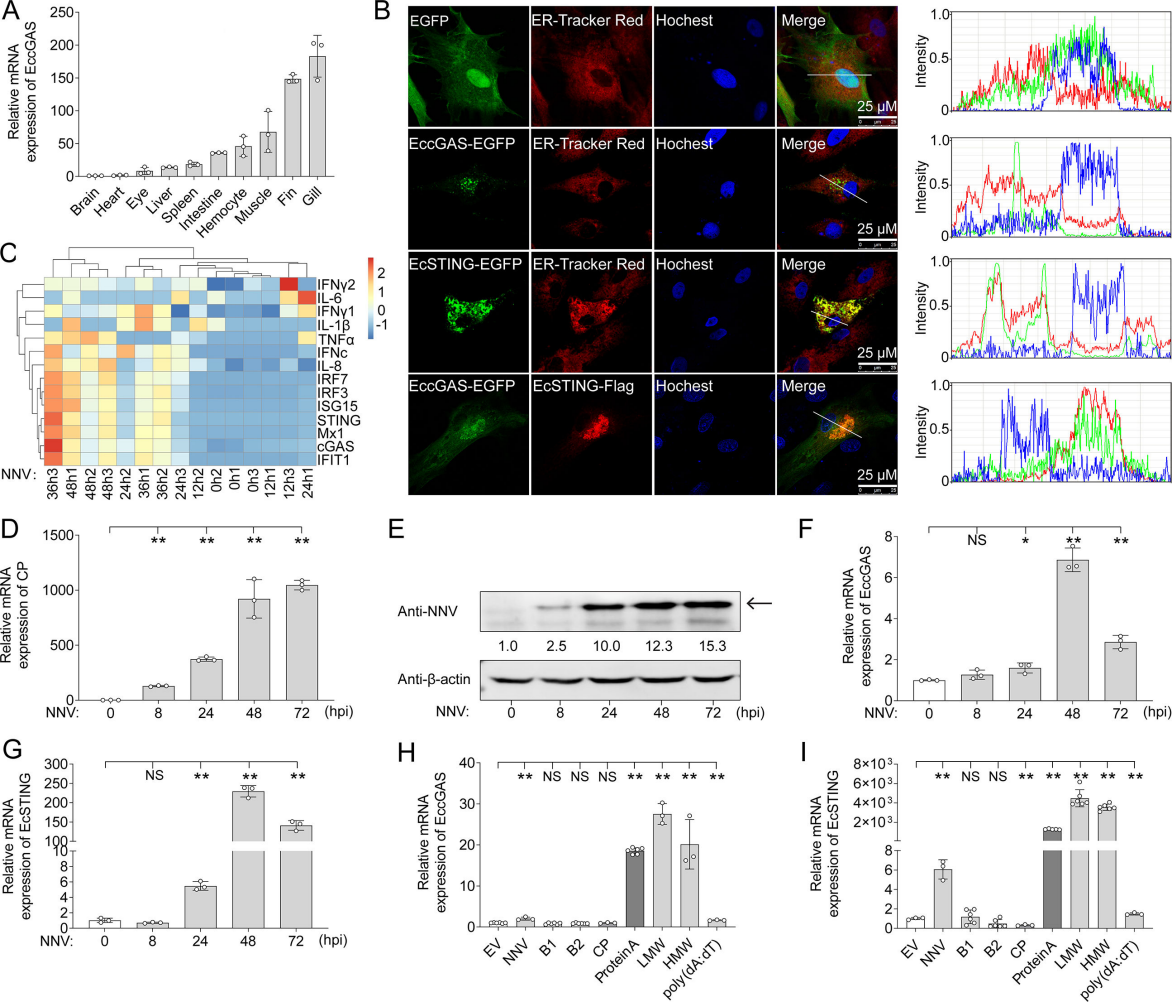
NNV infection upregulates EccGAS and EcSTING expression. (**A**) Tissue distribution of EccGAS analyzed by qRT-PCR. (**B**) The subcellular localization of EccGAS or EcSTING. Samples were observed under ﬂuorescence microscopy, and the fluorescent intensity on the white line was analyzed for co-localization. (**C**) The heatmap of immune-related genes in the EccGAS-mediated IFN signaling pathway from the transcriptome data of OGNNV-infected GB cells. (**D and E**) The expression profiles of CP during OGNNV infection. The mRNA level of CP (**D**) was detected by qRT-PCR, while the protein level of CP (**E**) was determined by Western blotting. The expression fold changes are shown. (**F and G**) The mRNA level of EccGAS or EcSTING in OGNNV-infected GB cells was detected by qRT-PCR. The value at 0 h [phosphate-buffered saline (PBS) mock stimulation] was set as 1. (**H and I**) The mRNA levels of EccGAS and EcSTING in GB cells with different treatments were measured by qRT-PCR. *P < 0.05, **P < 0.01. NS, no significance.

The abundance of *EccGAS* in health grouper gill and fin (Fig. 1A) was demonstrated by quantitative reverse transcript-PCR (qRT-PCR). Fluorescent results of overexpression showed that EccGAS co-localized with EcSTING at the endoplasmic reticulum in both grouper brain (GB) cells (Fig. 1B) and fathead minnow (FHM) cells (Fig. S1). To conﬁrm whether the cGAS-STING signaling pathway was involved in anti-NNV reaction, the transcriptome data generated from OGNNV-infected GB cells were analyzed. It was found that the expression levels of EccGAS, EcSTING, IRF3/IRF7, IFNc, and ISGs were upregulated after viral infection (Fig. 1C), indicating a potential anti-RNA virus role of EccGAS-mediated IFN signaling in response to NNV infection. To further explore the roles of EccGAS and EcSTING in the anti-viral response, qRT-PCR was performed to investigate their expression patterns treated with different stimuli, including NNV infection, overexpression of four viral proteins (B1, B2, CP, and ProA), or incubation of low molecular weight-/high molecular weight- polyinosinic-polycytidylic acid [LMW-/HMW-poly(I:C)] and poly(deoxyadenylic-deoxythymidylic) acid [poly(dA:dT)]. To broaden the scope of this study, the contentious B1 was also included as a stimulus. During OGNNV replication (Fig. 1D and E), the mRNA levels of EccGAS or EcSTING were upregulated after 24 h, peaking at 48 h (Fig. 1F and G). Furthermore, both EccGAS (Fig. 1H) and EcSTING (Fig. 1I) were remarkably upregulated after NNV infection, poly(dA:dT), ProA, or LMW-/HMW-poly(I:C) treatments, with the latter three being the most responsive. The putative B1, like B2, did not cause significant variation of cGAS expression (Fig. 1H and I). These results suggest that EccGAS and EcSTING can respond to NNV infection.

### EccGAS or EcSTING suppresses OGNNV replication in GB cells

**Fig 2:**
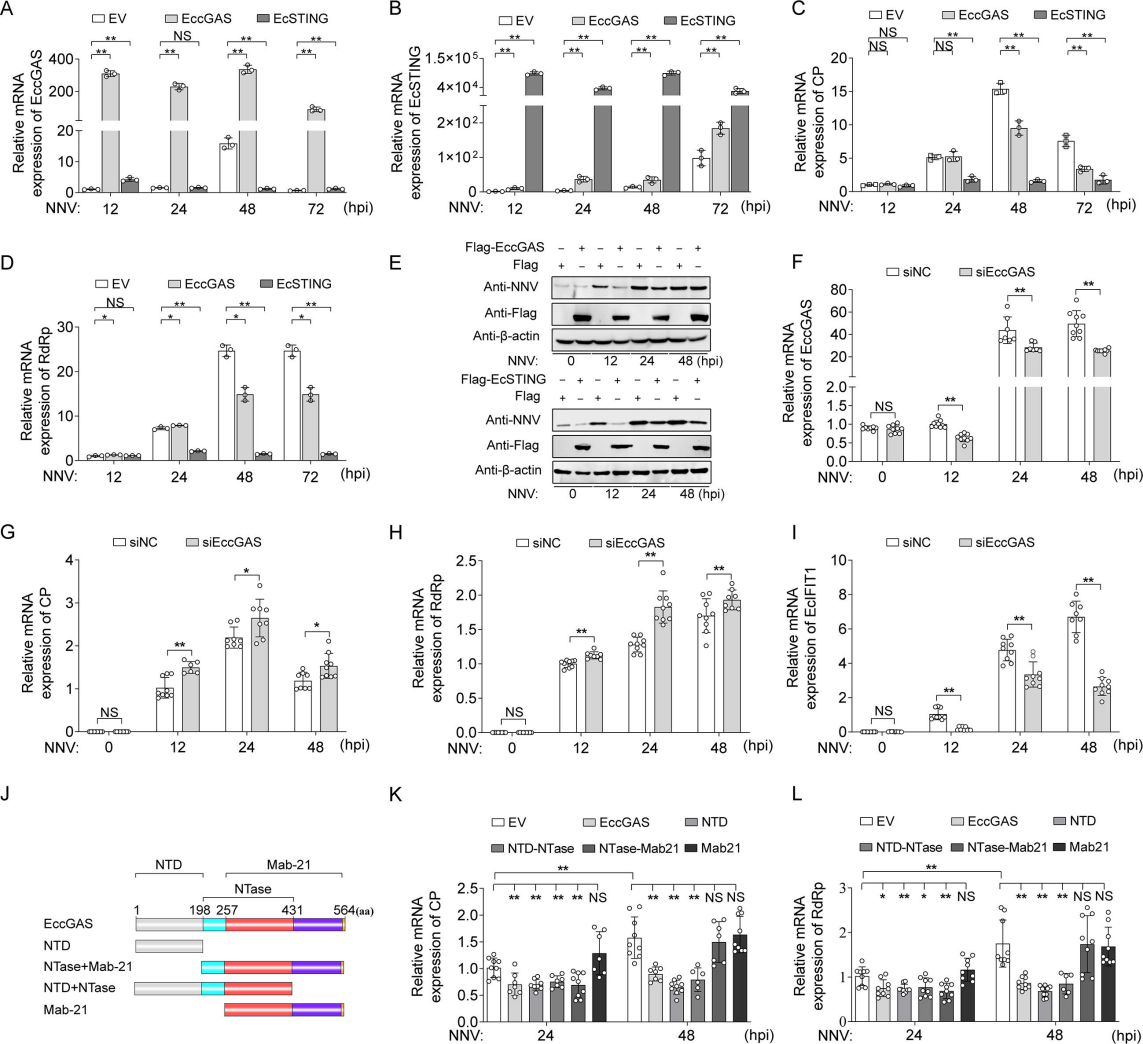
EccGAS or EcSTING inhibit OGNNV replication in GB cells. (**A–D**) The mRNA level of EccGAS (**A**), EcSTING (**B**), NNV CP (**C**), and RdRp (**D**) in OGNNV-infected GB cells transfected with the indicated expressing plasmids was measured by qRT-PCR. (**E**) The protein levels of NNV CP in OGNNV-infected GB cells transfected with the indicated expressing plasmids were determined by Western blotting. (**F–I**) The mRNA level of EccGAS (**F**), NNV CP (**G**), and RdRp (**H**), EcIFIT1 (**I**) in OGNNV-infected GB cells transfected with siRNAs against control (siNC) or EccGAS were detected by qRT-PCR. (**J**) The schematic diagram of truncated EccGAS mutants. (**K and L**) The mRNA level of NNV CP (**K**) and RdRp (**L**) in OGNNV-infected GB cells transfected with the indicated expressing plasmids was examined by qRT-PCR. *P < 0.05, **P < 0.01. NS, no significance.

To reveal the anti-viral functions of EccGAS and EcSTING, the OGNNV challenge was performed after overexpression of Flag-EccGAS or Flag-EcSTING for 48 h in GB cells (Fig. 2A and B). EccGAS induced the expression of EcSTING (Fig. 2B). The overexpression of EccGAS or EcSTING significantly suppressed OGNNV replication as indicated in the mRNA levels of CP and RdRp (Fig. 2C and D) and the protein level of CP (Fig. 2E). In contrast, short interfering RNA (siRNA)-mediated EccGAS knockdown (Fig. 2F) promoted the mRNA levels of OGNNV CP and RdRp and inhibited the expression of EcIFIT1 (Fig. 2G through I), an ISG, suggesting that both EccGAS and EcSTING were involved in the anti-NNV effect.

To identify the specific structural domains within EccGAS responsible for its anti-viral activity, four EccGAS truncated mutants were constructed (Fig. 2J) and overexpressed before OGNNV infection. The resulting expression of CP and RdRp revealed that both the N-terminal domain (NTD, amino acid residues 1–198) and the nucleotidyltransferase (NTase) domain (residues 199–431), but not the Mab-21 domain (residues 258–564), were required for the anti-NNV function of EccGAS (Fig. 2K and L). These data suggest that the NTD and NTase domains of EccGAS play primary roles in suppressing NNV replication.

### The EccGAS-STING pathway axis regulates the expression of IRF3/IRF7-IFNc-ISGs to resist NNV replication

**Fig 3:**
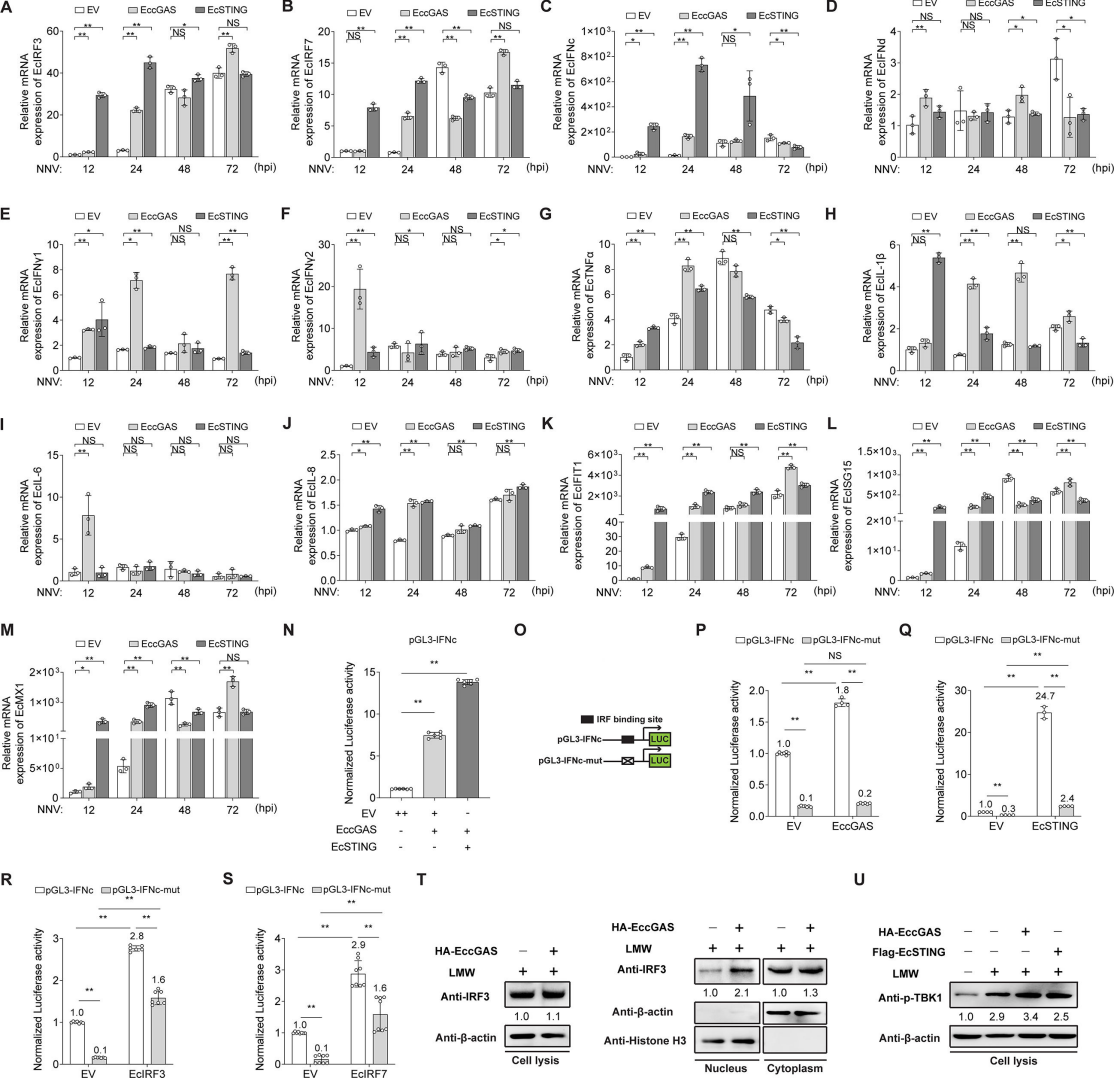
The EccGAS-STING axis activates EcTBK1 and regulates the expression of EcIRF3/EcIRF7-IFNc-ISGs to resist NNV infection in GB cells. (**A–M**) The mRNA level of EcIRF3 (**A**), EcIRF7 (**B**), EcIFNc (**C**), EcIFNd (**D**), EcIFNγ1 (**E**), EcIFNγ2 (**F**), EcTNF-α (**G**), EcIL-1β (**H**), EcIL-6 (**I**), EcIL-8 (**J**), EcIFIT1 (**K**), EcISG15 (**L**), and EcMx1 (**M**) in OGNNV-infected GB cells transfected with the indicated expressing plasmids was measured by qRT-PCR. (**N**) Luciferase activity analysis of *EcIFNc* promoter in GB cells transfected with the indicated expressing plasmids. (**O**) The schematic representation of IRF site mutations on the *EcIFNc* promoter. (**P–S**) Luciferase activity analysis of *EcIFNc* or its mutant (*EcIFNc*-mut) promoter in GB cells transfected with EccGAS, EcSTING, EcIRF3, or EcIRF7. (**T**) The protein levels of EcIRF3 in whole cell lysis, cytoplasm, and nucleus fraction of EccGAS overexpressed and LMW-poly(I:C)-stimulated GB cells were detected by Western blotting. (**U**) Immunoblot analysis of the phosphorylated EcTBK1 in EccGAS or EcSTING overexpressed and LMW-poly(I:C)-stimulated GB cells. The expression fold changes are shown under the indicated protein. *P < 0.05, **P < 0.01. NS, no significance.

To illustrate the downstream signal pathway regulated by EccGAS-EcSTING, OGNNV infection was conducted in EccGAS or EcSTING overexpressed GB cells. In response to OGNNV, EccGAS or EcSTING upregulated the expression levels of EcIRF3, EcIRF7, and EcIFNc but only slightly affected EcIFNd, EcIFNγ1, and EcIFNγ2 expressions compared to EcIFNc (Fig. 3A through F). The expression levels of ISGs, such as EcIFIT1, EcISG15, and EcMx1, were significantly upregulated (Fig. 3K through M). However, OGNNV infection and overexpression of EccGAS or EcSTING only slightly upregulated the proinflammatory cytokines (EcTNF-α, EcIL-1β, EcIL-6, and EcIL-8) compared with ISGs (Fig. 3G through J). In luciferase activity assays, EccGAS, EcSTING, EcIRF3, or EcIRF7 activated EcIFNc promoter activity (Fig. 3P through S). Furthermore, co-transfection of EccGAS and EcSTING enhanced the activity of the EcIFNc promoter compared with EccGAS, suggesting that EccGAS and EcSTING have synergistic effects in regulating EcIFN promoter activity (Fig. 3N). However, their regulatory effects rely on the IRF site on the EcIFNc promoter as proven by the reduction of luciferase activity in the mutated promoter (Fig. 3P through S). In addition, overexpression of EccGAS in response to low-molecular weight (LMW) stimulation promoted the nuclear translocation of EcIRF3 and the phosphorylation of EcTBK1, suggesting that EccGAS could activate EcTBK1 and EcIRF3 (Fig. 3T and U). We also observed that OGNNV infection induced the nuclear translocation of EcIRF3 and EcIRF7 in GB cells (Fig. S2). To sum up, the EccGAS-STING axis activates EcTBK1 and regulates the expression of EcIRF3/EcIRF7-EcIFNc-ISGs, rather than proinflammatory cytokines, to resist NNV infection.

### NNV proteins regulate EccGAS-mediated IFN response in a two-way manner

**Fig 4:**
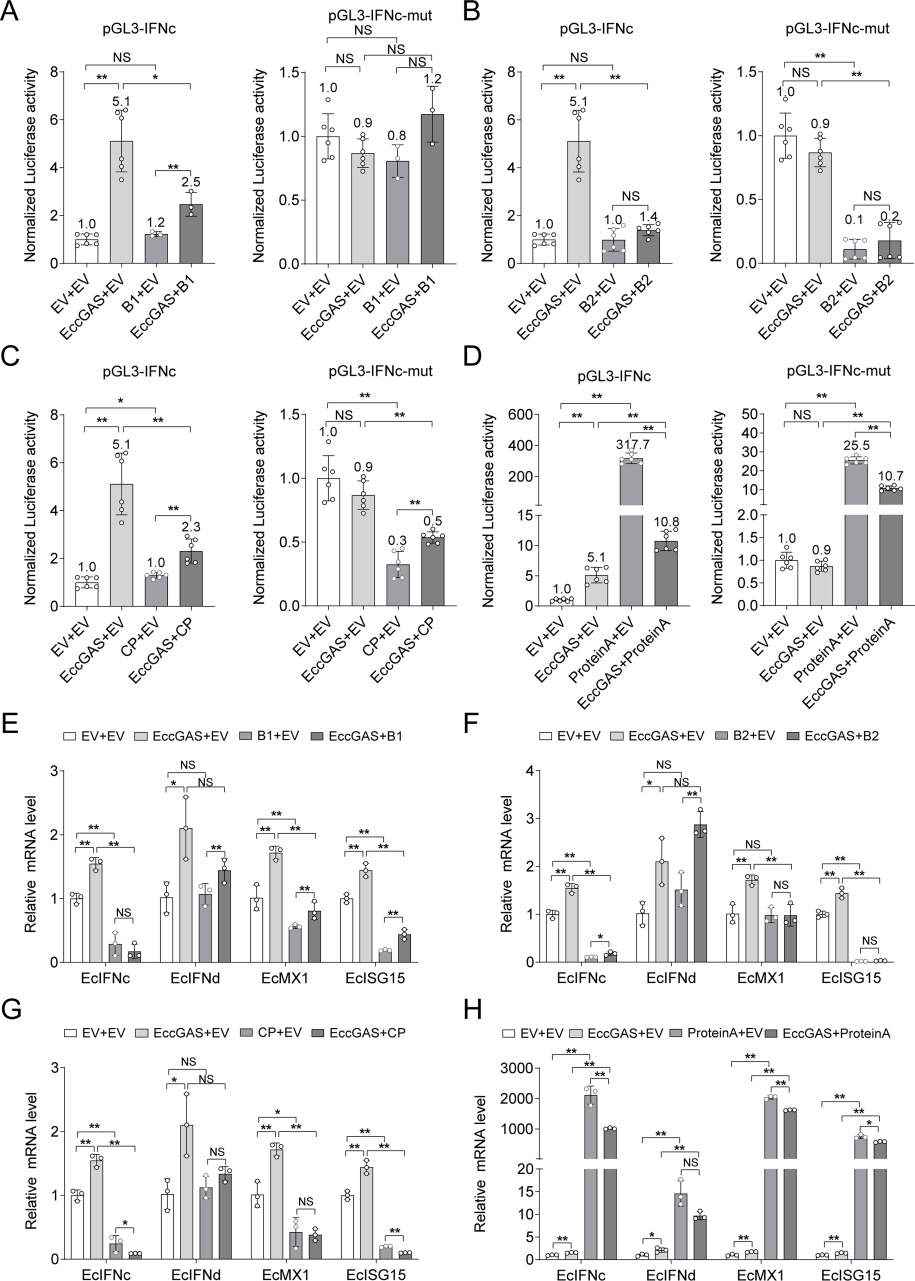
NNV proteins regulate EccGAS-mediated IFN response. (**A–D**) Luciferase activity of *EcIFNc* or its mutant promoter in GB cells co-transfected with hemagglutinin (HA) -EccGAS and Flag-tagged NNV protein plasmid. (**E–H**) The mRNA levels of EcIFNc, EcIFNd, EcMX1, and EcISG15 in GB cells co-transfected with HA-EccGAS and Flag-tagged NNV proteins were measured using qRT-PCR. *P < 0.05, **P < 0.01. NS, no significance.

To explore the influence of the NNV proteins on the EccGAS-mediated IFN response, individual viral proteins, including the contentious B1, were co-expressed with EccGAS in GB cells. NNV B1, B2, and CP significantly reduced the EcIFNc promoter activity activated by EccGAS (Fig. 4A through C) and the expression of EcIFNc, EcMx1, and EcISG15 induced by EccGAS (Fig. 4E through G). In contrast, the co-expression of NNV ProA and EccGAS significantly upregulated the EcIFNc promoter activity (Fig. 4D) and the expression levels of EcIFNc, EcMx1, and EcISG15 (Fig. 4H). These results suggest that NNV-encoded proteins can realize two-way regulation of EccGAS-mediated IFN signaling. Likewise, EccGAS may be a critical target for the dynamic regulation of EcIFNc and ISGs necessary for NNV to achieve immune escape.

### EccGAS interacts with NNV CP and ProA

**Fig 5:**
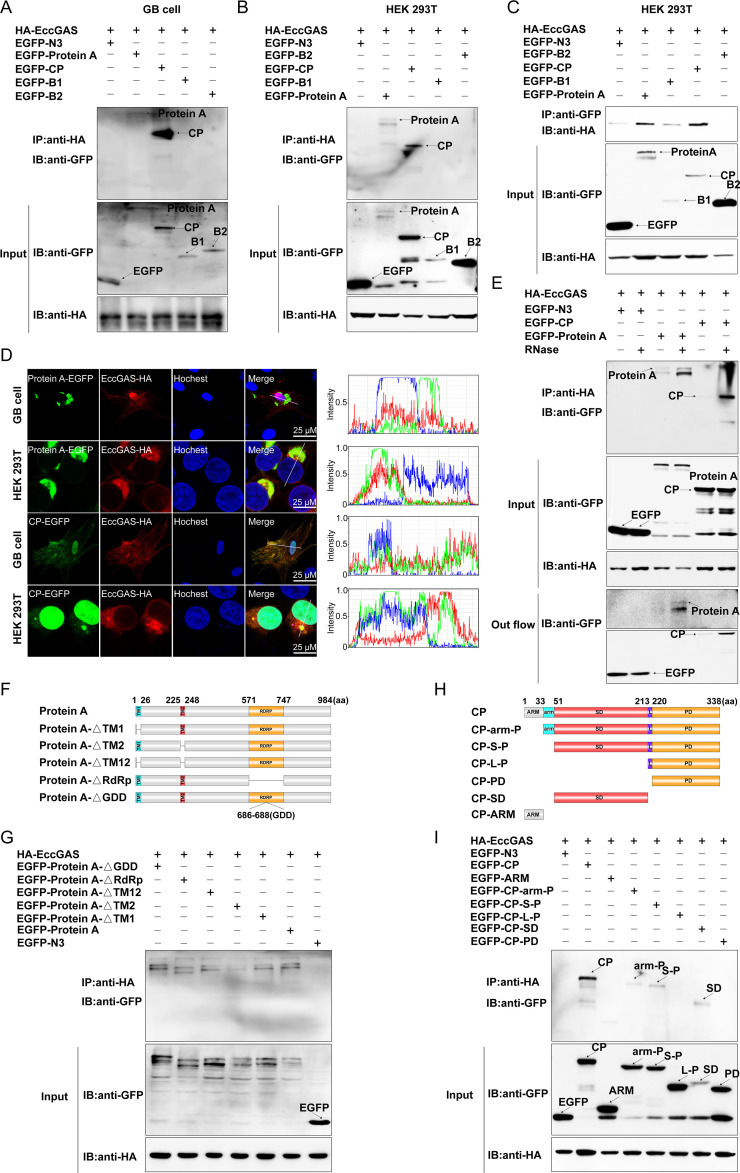
EccGAS interacts with both CP and ProA. (**A–C**) Co-IP assays of NNV proteins and EccGAS in indicated cells. (**D**) Co-localization of EccGAS and ProA or CP. (**E**) Co-IP assays of NNV proteins and EccGAS in the presence of RNase. (**F**) Schematic diagram of ProA and its deleted mutants. (**G**) Co-IP assays of EccGAS and ProA or its deletion mutants. (**H**) Schematic diagram of CP and its deleted mutants. (**I**) Co-IP assays of EccGAS and CP or its mutants. IP, immunoprecipitation; IB, immunoblot.

To evaluate the potential interaction between NNV proteins and EccGAS, co-immunoprecipitation (Co-IP) assays were conducted in both GB or HEK 293T cells. As shown in Fig. 5A and B, GFP-ProA and GFP-CP could be precipitated by HA-EccGAS using anti-HA magnetic beads. Similarly, HA-EccGAS could also be pulled down by GFP-ProA and GFP-CP using anti-GFP magnetic beads (Fig. 5C), confirming that ProA and CP could interact with EccGAS. Subsequently, immunofluorescence results showed that ProA or CP co-localized with EccGAS in the cytoplasm (Fig. 5D). Furthermore, RNase was added to the Co-IP assays to eliminate the possible RNA bridge in the protein interaction. The interactions between ProA or CP and EccGAS were all enhanced after RNase addition, suggesting that RNA may not play a bridging role in these interactions (Fig. 5E). To further determine which domain of ProA was required for the interaction with EccGAS, various GFP-tagged motif-deleted mutants of ProA were constructed (Fig. 5F) for domain mapping. Co-IP results demonstrated that the GDD domain, RdRp domain, or TM12 domain of ProA was required for binding with EccGAS (Fig. 5G). A domain mapping of CP interacting with EccGAS was also carried out. A series of plasmids of GFP-tagged truncated CP mutants were constructed (Fig. 5H) and co-transfected with HA-EccGAS. As shown in Fig. 5I, EccGAS interacted with CP-arm-P, CP-S-P, and CP-SD but not with CP-ARM, CP-L-P, or CP-PD. These results indicate that NNV CP or ProA can directly bind EccGAS through protein-protein interaction.

### NNV CP and ProA dynamically regulate the degradation of EccGAS via the ubiquitin pathway

**Fig 6:**
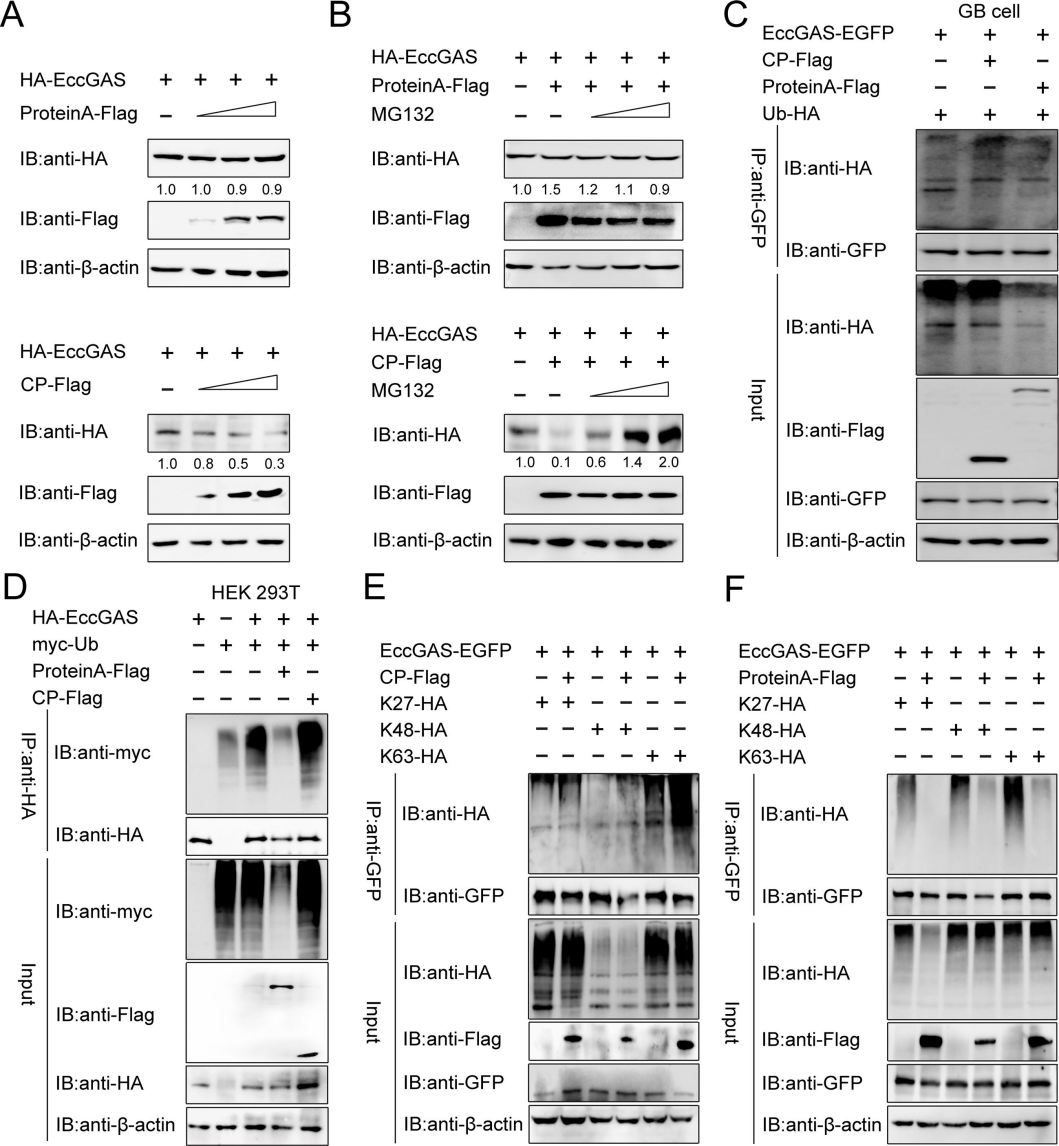
CP induces while ProA restrains the polyubiquitination of EccGAS. (A and B) Summary of Western blotting analysis of HEK 293T cells co-transfected with HA-EccGAS and ProA-Flag or CP-Flag plasmids. The protein contents of EccGAS when co-transfected with HA-EccGAS and ProA-Flag or CP-Flag plasmids were detected by Western blotting in HEK 293T cells without (**A**) or with (**B**) MG132 treatment. (**C and D**) The polyubiquitination of EccGAS with additional wild-type ubiquitin in GB cells (**C**) and HEK 293T cells (**D**) transfected with ProA-Flag or CP-Flag plasmids was detected by Western blotting. (E and F) The polyubiquitination of EccGAS with different additional ubiquitin mutants as indicated in HEK 293T cells transfected with CP-Flag or ProA-Flag plasmids was detected by Western blotting.

As both CP and ProA bound to EccGAS, their relationship at the protein level was investigated in GB cells and HEK 293T cells. We first monitored the variation of EccGAS protein content co-expressed with NNV ProA or CP. CP overexpression reduced EccGAS content in a dose-dependent manner, whereas ProA overexpression did not affect the EccGAS expression (Fig. 6A). In addition, MG132 (a proteasome inhibitor) was used to determine whether the ubiquitin-proteasome pathway was responsible for EccGAS reduction. As shown in Fig. 6B, MG132 could block the CP-induced reduction of EccGAS protein while not affecting the protein content of EccGAS in ProA samples, indicating that EccGAS undergoes CP-mediated proteasomal degradation. Second, ubiquitination assays in both GB and HEK 293T cells with MG132 were performed. EccGAS itself was found to be polyubiquitinated in the presence of wild-type ubiquitin (Ub-HA or myc-Ub). Interestingly, the polyubiquitination of EccGAS was enhanced when co-expressing with CP and was inhibited in ProA co-expressing samples (Fig. 6C and D). Third, different ubiquitin mutants with a single active lysine residue were expressed in ubiquitination assays to identify the ubiquitin linkage types. The results indicated that CP induced the polyubiquitination of EccGAS with K48 and K63 linkages (Fig. 6E). In contrast, ProA inhibited these three forms of ubiquitination of EccGAS (Fig. 6F). In conclusion, CP induces the polyubiquitination of EccGAS with K48 and K63 linkages, and ProA restrains this reaction.

### EcUBE3C participated in CP-assisted EccGAS polyubiquitination

**Fig 7:**
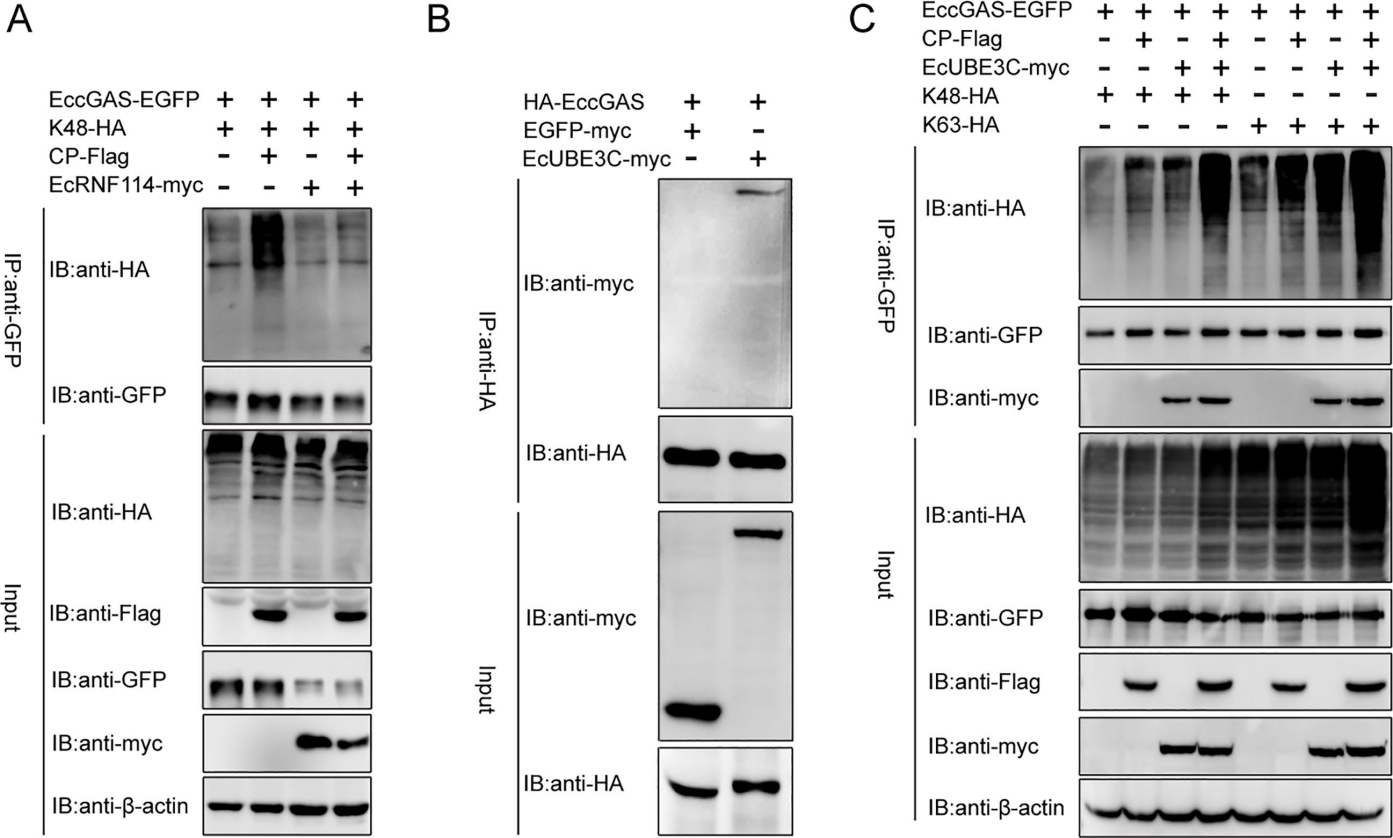
EcUBE3C participates in CP-assisted EccGAS ubiquitination. (**A**) The polyubiquitination of EccGAS in HEK 293T cells with K48-ubiquitin in the presence of EcRNF114 and CP was detected by Western blotting. (**B**) Co-IP assays of EccGAS and EcUBE3C. (**C**) The polyubiquitination of EccGAS in HEK 293T cells transfected with EcUBE3C-myc and CP-Flag plasmids was detected by Western blotting.

Since CP does not catalyze ubiquitination, we hypothesized that other E3 ubiquitin ligases may be involved in the polyubiquitination of EccGAS with the help of CP. To verify this hypothesis, two potential ligases were selected. EcRNF114 is the homolog of LjRNF114 that was involved in CP-assisted ubiquitination (39). EcUBE3C is the homolog which participates in cGAS polyubiquitination (40). As shown in Fig. 7A, EcRNF114 did not promote K48-linked EccGAS ubiquitination in the presence of CP. In contrast, EcUBE3C not only interacted with EccGAS (Fig. 7B) but also enhanced the polyubiquitination of EccGAS mediated by CP in both K48 and K63 linkages (Fig. 7C). All our results suggested that EcUBE3C catalyzes the CP-assisted K48- and K63-linked polyubiquitination of EccGAS and promotes the subsequent degradation.

## DISCUSSION

**Fig 8:**
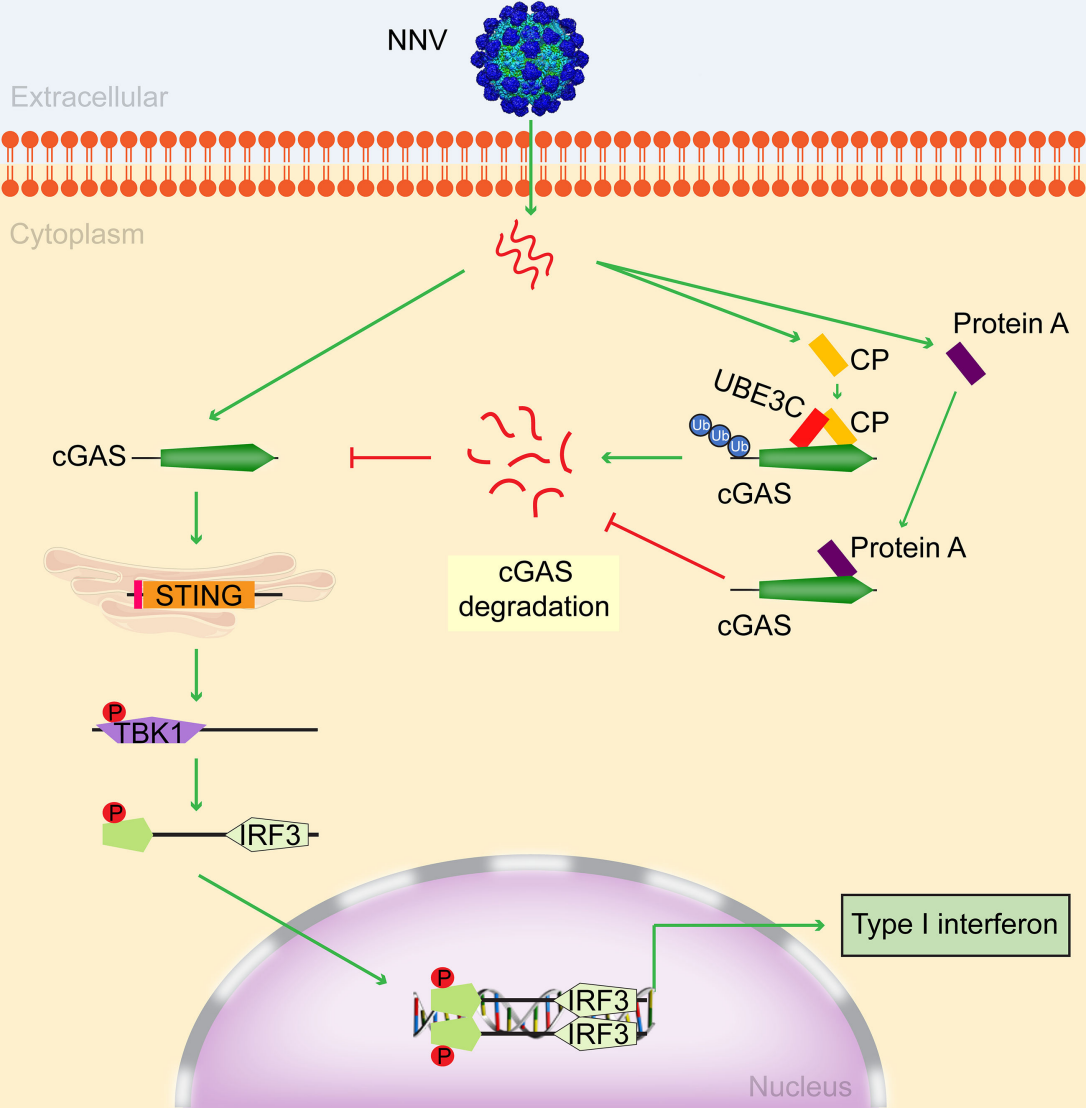
Schematic model of EccGAS regulation of NNV infection. Upon NNV infection, EccGAS can activate EcTBK1 and EcIRF3/EcIRF7 via EcSTING to induce the expression of IFNc and ISGs, leading to the EccGAS-mediated IFN response in the early stage. Simultaneously, NNV CP and ProA interact with EccGAS to dynamically regulate its protein content through the ubiquitin-proteasome pathway. Notably, NNV CP promotes the ubiquitination of EccGAS via EcUBE3C ubiquitin ligase. The abundance of CP in the mid-and late stages of NNV replication strongly inhibits the cGAS-STING-IRF3/IRF7 signaling pathway. These findings reveal a novel strategy for RNA viruses to evade cGAS-mediated innate surveillance.

The IFN-I-related signaling pathway plays a key role in the anti-viral immune response. Our recent results have already revealed that the RIG-I/MDA5-MAVS-IRF3 signaling pathway can trigger the activation of IFN in response to NNV infection (36). Herein, we reported another mechanism for the positive regulation of IFN production in response to NNV infection via the cGAS-STING-IRF3/IRF7 signaling pathway (Fig. 8). Specifically, in the context of NNV infection, upregulated EccGAS can activate EcTBK1 and EcIRF3/EcIRF7 via EcSTING to facilitate IFN-I activation and ISGs expression, leading to the viral inhibition. As a counterattack by the NNV, its proteins, CP and ProA, interact with EccGAS to dynamically regulate the protein levels of EccGAS through the ubiquitin-proteasome pathway. This study has revealed that cGAS, traditionally recognized as a cellular DNA receptor, can effectively inhibit aquatic RNA virus NNV replication by stimulating the host’s anti-viral immune response. Meanwhile, NNV CP was found to promote EccGAS protein degradation, while ProA hindered this degradation process. Collectively, viral proteins negatively influenced EccGAS-mediated innate immunity to achieve NNV replication. We have demonstrated that cGAS is one of the core targets for NNV-host interaction.

It is widely recognized that cGAS is a central component of the innate immune response to cytosolic DNA derived from diverse pathogens. However, cGAS as an RNA sensor has attracted increasing interest. There is evidence that cGAS is also engaged in the responses to RNA viruses. Accordingly, we found that orange-spotted grouper EccGAS was involved in the anti-viral response to NNV, an RNA virus. These findings suggest that cGAS shows anti-RNA virus activity in aquatic animals, similar to the findings of studies with mammals.

Many viruses have evolved mechanisms to evade cGAS-mediated innate immune responses by targeting cGAS. For example, the dengue virus NS2B protein can target cGAS and promote cGAS degradation (26). Kaposi’s sarcoma-associated herpesvirus’ periplasmic protein ORF52 inhibits type I interferon production by directly interacting with cGAS (41), and the herpes simplex virus 1 periplasmic protein VP22 interacts with cGAS and inhibits its enzymatic activity (42). In this study, we found that two NNV-encoded proteins, CP and ProA, could interact with EccGAS. The underlying mechanism involves CP binding EccGAS through the arm-P, S-P, and SD domains (Fig. 5I), and this promotes EccGAS degradation (Fig. 6A and B), leading to the inhibition of the EccGAS-induced IFN signaling pathway (Fig. 4C and G). Our previous research found that NNV ProA promoted the expression of type I interferon, which demonstrated an opposite function to that of CP (36). In this study, we found that ProA also promoted the EccGAS-mediated activation of the IFN signaling pathway (Fig. 4D and H) by interacting with EccGAS to inhibit its ubiquitination and degradation (Fig. 6A through D and F). K48-linked ubiquitination mediates proteasome degradation, while K63-linked ubiquitination is associated with innate immune signaling (43). We found that CP promoted K48- and K63-linked ubiquitination of EccGAS (Fig. 6E), whereas ProA inhibited these ubiquitination procedures (Fig. 6F). We hypothesized that NNV may target EccGAS via CP or ProA to dynamically regulate the IFN signaling pathway through the ubiquitin-proteasome pathway to create an environment conducive to NNV replication, which ultimately leads to the successful proliferation of NNV. The E3 ubiquitin ligase is the determinant for the specific target undergoing the ubiquitin-proteasome pathway. It has been shown that CP interacts with the E3 ubiquitin ligase RNF34 to promote the degradation of IRF3 (44) and also that it interacts with the E3 ubiquitin ligase RNF114 to promote the degradation of TRAF3 (37). Tegument protein UL21 of alpha-herpesvirus interacts with the E3 ubiquitin ligase UBE3C to promote the degradation of cGAS (40). Here, we found that the E3 ubiquitin ligase UBE3C interacted with CP (Fig. 7B) and was responsible for CP-induced EccGAS ubiquitination rather than EcRNF114 (Fig. 7A and C).

Although cGAS restricts the replication of some RNA viruses, it is not required for the RNA virus-induced type I interferon response. In mammals, although cGAS responds to RNA virus infection, the response may be indirect. Although related reports suggest that cGAS could bind dsRNA, this interaction did not induce the production of cGAMP (4). In addition, cGAS deficiency did not affect the production of SeV-induced IFN-β (45). Therefore, although cGAS restricts the replication of some RNA viruses, it is not required for the RNA virus-induced type I interferon response. A recent study reported that DENV infection led to mitochondrial damage and the release of mitochondrial DNA into the cytoplasm, which then activated the cGAS-STING pathway and enhanced the host defense response (28). This finding suggests the possibility that cGAS may play an indirect role in limiting RNA virus infection. It has also been shown that STING was capable of interacting with RIG-I and MAVS, which were key components of the RNA sensing pathway, suggesting that it may play a role in the production of cytokines induced by RNA viruses (12). We found that the addition of RNase did not affect the binding of EccGAS to NNV CP or ProA, suggesting that RNA may not play a bridging role during their interaction. Our data showed that cGAS can be activated by RNA viruses to induce the expression of type I interferon. However, whether EccGAS responds directly or indirectly to the viral RNA genome needs to be further investigated.

There is a current debate concerning the regulatory role of cGAS in innate immunity. Multiple findings in mammals suggest that cGAS can exert an effective anti-viral effect by inducing the production of type I interferon (3, 5, 46). In fish, cGAS-mediated response is more complex, and cGAS can suppress IFN signaling. Zhou *et al.* found that grass carp cGAS could target STING to inhibit IFN production (29). Similarly, crucian carp cGAS and grass carp cGAS could negatively regulate the IFN response by interacting with the dsRNA sensor RIG-I that promotes spring viremia of carp virus replication (30). Likewise, the cGAS of orange-spotted grouper interacts with STING to inhibit the production of IFN and ISG and promotes the replication of Singapore grouper iridovirus (an aquatic DNA virus) (47). However, other fish cGASs exert a positive IFN response involved in anti-viral effects. Xu et al. found that there were two homologous cGAS proteins in grass carp, cGASa, and cGASb that exhibited opposite effects on the regulation of IFN. The cGASa interacted with STING to activate IRF7-induced IFN production and thereby promote resistance to GCRV, while cGASb could compete with cGASa to bind STING to negatively regulate IFN responses to favor GCRV replication (31). Our research results were consistent with the function of grass carp cGASa, as we found that orange-spotted grouper cGAS could activate the phosphorylation of TBK1 and promote the nuclear translocation of IRF3 that, in turn, induced the production of IFNs and ISGs to participate in the NNV resistance. Orange-spotted grouper cGAS showed different modes of IFN regulation in response to different viral infections, and these differences need to be further investigated.

In conclusion, our study demonstrated that cGAS, an enzyme that is widely recognized as a DNA PRR, exhibits a novel manner of anti-viral response to an aquatic RNA virus, NNV, via IFN activation. The viral proteins CP and ProA target cGAS to dynamically modulate IFN signaling, which is one of the immune evasion patterns employed by NNV. These findings not only enrich our knowledge of the mechanism of NNV-host interaction but also provide a theoretical basis for understanding the complex processes of viral infection and pathogenesis. Our data will be useful for target screening and the development of immunological agents against NNV.

## MATERIALS AND METHODS

### Fish maintenance and tissue collection

Healthy orange-spotted groupers, averaging 10 g in body weight and 8 cm in body length, were purchased from Foshan fish farm (Guangdong, China) and kept in a seawater recirculating aquaculture system for 1 week before the experiment. For tissue distribution assay, healthy orange-spotted grouper tissues including brain, heart, eye, liver, spleen, intestine, hemocyte, muscle, fin, and gill were collected from six individuals and stored as small pieces in RNAlater (Invitrogen).

### Cell lines and virus

GB cells established from the brain tissue of the *Epinephelus coioides* were maintained at 28°C with 5% CO_2_ in Dulbecco’s modified Eagle’s medium (DMEM) supplemented with 10% (vol/vol) fetal bovine serum (FBS) (Hyclone). HEK-293T cells were grown at 37°C with 5% CO_2_ in DMEM supplemented with 10% FBS. SC1 was obtained by subcloning the SSN-1 cell line by limited dilution in our laboratory and was cultured in L15 medium supplemented with 10% FBS at 27°C. The FHM cells were cultured and maintained in M199 medium supplemented with 10% FBS at 27°C. OGNNV-HN1 (GenBank accession numbers MG874757.1 and MG874758.1) was isolated from moribund orange-spotted grouper larvae collected in the Hainan province of China and propagated in SC1 cells as previously described (48). In the OGNNV infection experiment, GB cells were seeded into six-well plates for 12 h to 90% confluence and infected with OGNNV at a multiplicity of infection (MOI) of 2. After 1 h of adsorption at 28°C, the inoculum was removed; fresh DMEM plus 5% FBS was added; and GB cells were collected at the indicated time point post-infection.

### Antibodies and reagents

Antibodies including anti-HA (1:3,000; 3063R, Dia-An Biotech), anti-GFP (1:3,000; 3057, Dia-An Biotech), anti-Flag (1:3,000; 3064, Dia-An Biotech), anti-myc (1:3,000; 3097, Dia-An Biotech), anti-p-TBK1 (1:1,000; 5483S, Cell Signaling), anti-β-actin (1:20,000; 66009–1-Ig, Proteintech), anti-histone-H3 (1:2,000; 4499s, Cell Signaling), and the secondary antibodies of anti-rabbit IgG (H + L) (1:5,000; W4011, Promega, USA) and Donkey anti-rabbit IgG (H + L) Alexa Fluor 594 (1:1,000; A21207, ThermoFisher, USA) were used. Anti-IRF3 was prepared by GL Biochem (Shanghai) Ltd using two peptides (SNGRVHGDPSVWK and SKFDRNKQPQEISKL) derived from the EcIRF3 protein as antigens. Polyclonal antibody against NNV was prepared by our laboratory using the virus-like particle of OGNNV as the antigen (49).

The polyinosinic-polycytidylic acid [poly(I:C)], including high molecular weight (tlrl-pic) and LMW (tlrl-picw), as well as poly(dA:dT) (tlrl-patn), were purchased from InvivoGen. MG132 (S2619) was purchased from Selleck and dissolved in dimethyl sulfoxide (DMSO). Anti-GFP magnetic beads (SM038001) were purchased from Smart-life Sciences. Anti-HA magnetic beads (88836) were purchased from ThermoFisher.

### Plasmid

Three new vectors, pN3-Flag, pN3-myc, and pN3-HA, were constructed by replacing the GFP of pEGFP-N3 with a Flag, myc, or HA tag according to a previous study (36). The ORFs of EccGAS (accession number KT313003.1), EcSTING (accession number OR940901), EcIRF3 (accession number JX975468.1), EcIRF7 (accession number GU198923.1), EcRNF114 (accession number PP708720), and EcUBE3C (accession number PP708719) were cloned into pEGFP-N3, pN3-Flag vector, and/or pN3-HA. pEGFP-ProA and truncated mutants of ProA with EGFP-tag were prepared in our previous studies (36). pEGFP-B1, pEGFP-B2, pEGFP-CP, and truncated mutants of CP with EGFP-tag were prepared in our laboratory. cGAS mutations, including NTD, NTD + Ntase, Ntase + Mab21, and Mab21, were generated by PCR using specific primers based on the cGAS-Flag recombinant plasmid. The firefly luciferase reporter vectors of EcIFNc promoter (pGL3-EcIFNc) and its mutant (deletion of the IRF binding site of “CAGAAACTGAAAC”, pGL3-EcIFNc-mut) were constructed by cloning the promoter sequence of EcIFNc into pGL3-Basic (Fig. S2). pRL-CMV plasmids were purchased from Beyotime Company. The vectors of HA-tagged ubiquitin-WT (pCDEF-HA-Ub) and K27-HA, K48-HA, and K63-HA plasmids were provided in our laboratory. All recombinant plasmids were confirmed by DNA sequencing. The primer sequences are listed in Table S1.

### qRT-PCR

For all the collected samples, total RNA was extracted with RNeasy Mini Kit (Qiagen) and reverse transcribed into cDNA with PrimeScript II 1st Strand cDNA Synthesis Kit (Takara) according to the manufacturer’s instructions. Quantitative real-time PCR analysis was performed on a Light Cycler480 instrument (using the 384-well module) in a 10-µL volume containing 5 µL 2× Polarsignal qPCR mix (MIKX.MKG800, China), 1-µL cDNA template, 0.5-µL forward primer, 0.5-µL backward primer, and 3-µL double-distilled water. The PCR procedure of each assay in triplicate was used as follows: 40 cycles of amplification for 5 min at 95°C, 30 s at 95°C, 30 s at 60°C, and 15 s at 70°C. Data were normalized to the level of the β-actin gene in each sample using the 2^−ΔΔCt^ method. All real-time PCR primers are listed in Table S1.

### Expression patterns

In the stimulation assay, GB cells cultured in six-well plates were transfected with plasmids (μg) of pEGFP-B1, pEGFP-B2, pEGFP-CP, and pEGFP-ProA and immune stimulants (2 µg/mL) of LMW-/HMW-poly(I:C) and poly(dA:dT) using transfection reagent (MIKX, 11231804–01), and were collected at 24 h post-transfection. In the anti-viral experiment, GB cells were seeded into six-well plates for 12 h, transfected with plasmids (2 µg) of HA-EccGAS, its serial truncated mutants, HA-EcSTING, or EV (HA) for 24 h, infected with OGNNV (MOI = 2), and then collected at indicated time points. In the NNV protein regulation study, GB cells were transfected with EV or HA-EccGAS vectors, together with plasmids of B1-Flag, B2-Flag, CP-Flag, or ProA-Flag, and collected at 48 h post-transfection. All the collected samples were subjected to qRT-PCR for detection of the mRNA level or to Western blotting for evaluation of the protein level of indicated genes.

### RNA interferences

Three cGAS-specific siRNAs synthesized by RiboBio (China) were mixed in equal amounts as siEccGAS. GB cells cultured in six-well plates were transfected with siNC or siEccGAS by using Lipo8000 Transfection Reagent (Beyotime, C0533) with a final concentration of 50-nM siRNA per well. After 24 h post-transfection, GB cells were infected with OGNNV (MOI = 2). Finally, cell samples were collected at the time points indicated, and qRT-PCR experiments were performed to determine the viral copy number and gene expression. All siRNA sequences are listed in Table S1.

### Luciferase activity assay

In the activation assay of host factors, GB cells in 12-well plates were co-transfected with 1-µg EV (pN3-Flag) or vectors of Flag-EccGAS, Flag-EcSTING, EcIRF3-Flag, or EcIRF7-Flag, and a set of luciferase reporter vectors including the firefly vector (200 ng) pGL3-EcIFNc or pGL3-EcIFNc-mut and the internal control renilla vector (20 ng) pRL-CMV. In the activation assay of viral proteins, GB cells were co-transfected with EV (pN3-Flag) or HA-EccGAS plasmid together with the same amount of vectors of B1-Flag, B2-Flag, CP-Flag, or ProA-Flag in a total of 1 µg, and a set of luciferase reporter vectors. After 48 h post-transfection, cells were harvested and lysed in 200-µL Passive Lysis Buffer (Promega), and total cell lysates were subjected to determine the firefly and renilla luciferase activities with Dual-Luciferase Reporter Assay kit (Promega) according to the manufacturer’s instructions. The activity of luciferase was determined by Glomax (Promega).

### Co-immunoprecipitation, Western blotting, and nucleocytoplasmic separation

GB cells or HEK 293T cells were co-transfected with indicated plasmids and lysed with or without RNase at 48 h post-transfection. The cells were lysed at 4°C for 30 min with cell lysis buffer (Beyotime, p0013) supplemented with the protease inhibitor cocktail (Sigma-Aldrich, P8340-1ML) and then centrifuged at 13,000 × *g* for 5 min. The supernatants were subjected to Co-IP according to the manufacturer’s instructions for the specific antibodies or magnetic beads. The bound proteins from Co-IP or the total proteins from the collected cells with certain treatments were boiled in SDS-PAGE Protein Loading Buffer (Fdbio Science, FD006) for 10 min, separated on 10% SDS-PAGE gels, and then transferred to polyvinylidene fluoride (PVDF) membranes (Millipore). Membranes were blocked for 1 h at room temperature (RT) with 5% skimmed milk powders dissolved in tris-buffer saline with 0.1% Tween-20 (TBST) and were incubated with the indicated primary antibodies diluted with TBST according to the specific concentration at RT for 2 h or at 4°C overnight. The membranes were washed with TBST three times and incubated with horseradish peroxidase-conjugated secondary antibodies at RT for 1 h. The bands were visualized by the 5200 Chemiluminescence Imaging System (Tanon) after the membranes were washed three times with TBST. The gray values of the protein bands were analyzed using ImageJ software. In the activation assay of IRF3 or TBK1, GB cells were treated with 5-µg/mL LMW-poly(I:C) for 6 h after overexpression of EccGAS and EcSTING for 24 h. The nucleocytoplasmic separation was performed using the NE-PER Nuclear and Cytoplasmic Extraction Reagents (ThermoFisher, 78833) according to the manufacturer’s instructions, and then different fractions were subjected to Western blotting.

### Ubiquitination

GB cells or HEK 293T cells were transfected with plasmids of HA-EccGAS (HA-tagged or GFP-tagged), wild-type ubiquitin (myc-Ub or Ub-HA), ubiquitin mutants (K27-HA, K48-HA, or K63-HA), and EV (HA or myc), together with vectors of ProA-Flag, CP-Flag, EcRNF114-myc, or EcUBE3C-myc. At 36 h post-transfection, cells were treated with proteasomal inhibitor MG132 (20 mM) or DMSO for 6 h, respectively. The cell lysates were immunoprecipitated with anti-GFP magnetic beads or anti-HA magnetic beads. The immunoprecipitates were analyzed by Western blotting with indicated antibodies.

### Immunofluorescence assay

GB cells, FHM cells, or HEK 293T cells were seeded on 14-mm glass coverslips. After 16 h, cells were transfected with the indicated plasmids for 24 h. The coverslips were fixed with 4% paraformaldehyde (Biosharp, BL539A) for 10 min. Cells were permeabilized in 0.2% Triton X-100 (A110694-0100) for 15 min. After being washed three times with TBST, cells were blocked with 5% goat serum (BOSTER, AR0009) at RT for 1 h and then washed three times with TBST. The coverslips were incubated with the anti-Flag or anti-HA primary antibodies diluted with TBST for 2 h, washed three times with TBST, and incubated with the donkey anti-rabbit IgG (H + L) Alexa Fluor 594 diluted in TBST for 1 h. The endoplasmic reticulum and nuclei were counterstained with ER-Tracker Red (Beyotime, C1041) and Hoechst 33342 (ThermoFisher) according to the manufacturer’s instructions. Fluorescence microscopy (Leica TCS SP8 STED 3×; Leica, Germany) was employed to observe the samples, and Leica Application Suite (Advanced Fluorescence Lite version 2.8.0 build 7268) was used to analyze the fluorescent intensity for co-localization.

### Statistical analyses

All statistical analyses were performed using GraphPad Prism (GraphPad Software). Data are expressed as mean ± standard deviation. A bilateral unpaired Student *t*-test was used to calculate the statistical differences between the two groups. *P* < 0.05 was considered statistically significant. The asterisks indicated that the difference was statistically significant (*P < 0.05, **P < 0.01). NS means no significance. All experiments were performed three or more times independently.

## Data Availability

The full-length sequence of *Epinephelus coioides* EccGAS was deposited in GenBank under accession number KT313003.1.

## SUPPLEMENTAL MATERIAL

Supplemental material jvi.00686-24-s0001.docx Figures S1 to S3; Table S1.

ASM does not own the copyrights to Supplemental Material that may be linked to, or accessed through, an article. The authors have granted ASM a non-exclusive, world-wide license to publish the Supplemental Material files. Please contact the corresponding author directly for reuse.
