# Supplementary material for: Nervous necrosis virus capsid protein and Protein A dynamically modulate the fish cGAS-mediated IFN signal pathway to facilitate viral evasion
Source: J Virol. 2024 Jun 18;98(7):e00686-24. doi: 10.1128/jvi.00686-24 (PMC11264591; doi:10.1128/jvi.00686-24)
Supplement: Supplemental material — Figures S1 to S3; Table S1. [file jvi.00686-24-s0001.docx]

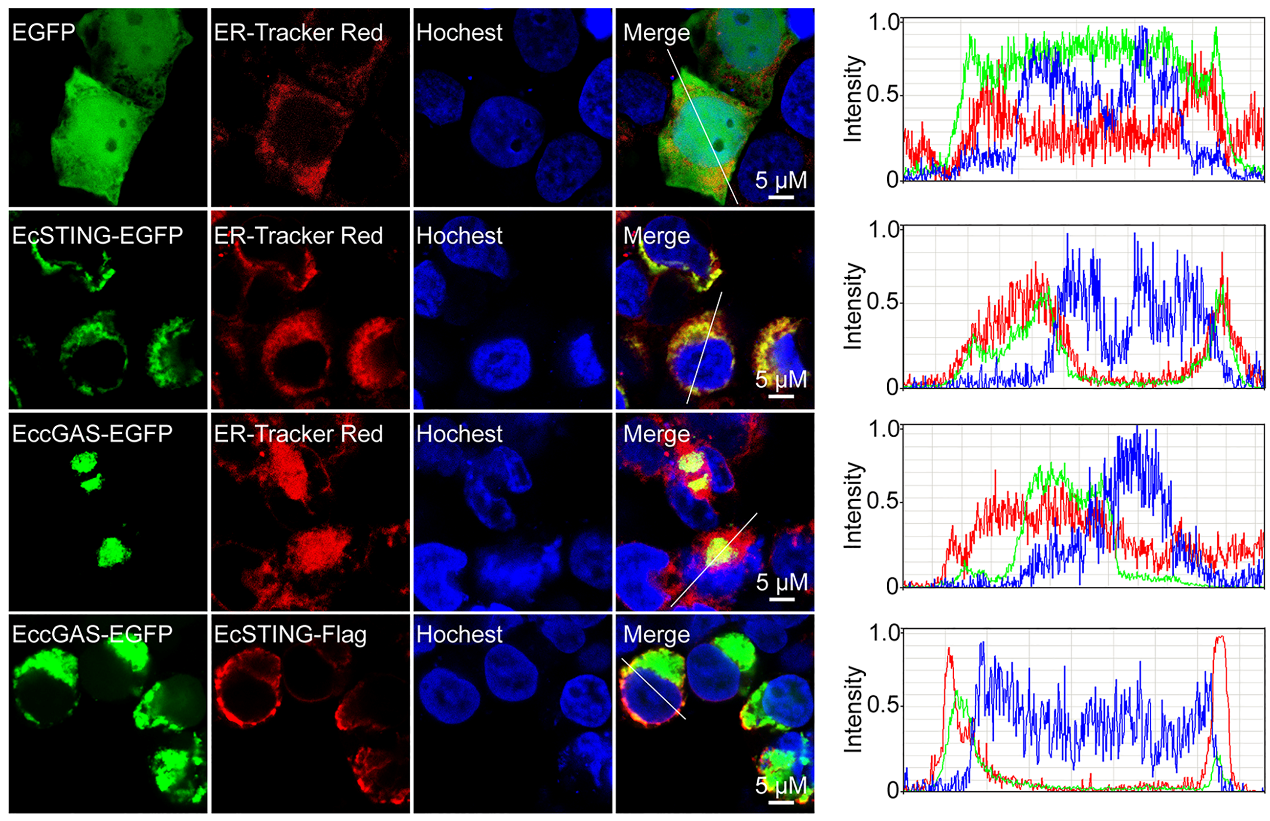


Fig. S1 The subcellular localization of EccGAS or EcSTING in FHM cells. Samples were observed under ﬂuorescence microscopy and the fluorescent intensity on the white line was analyzed for colocalization.


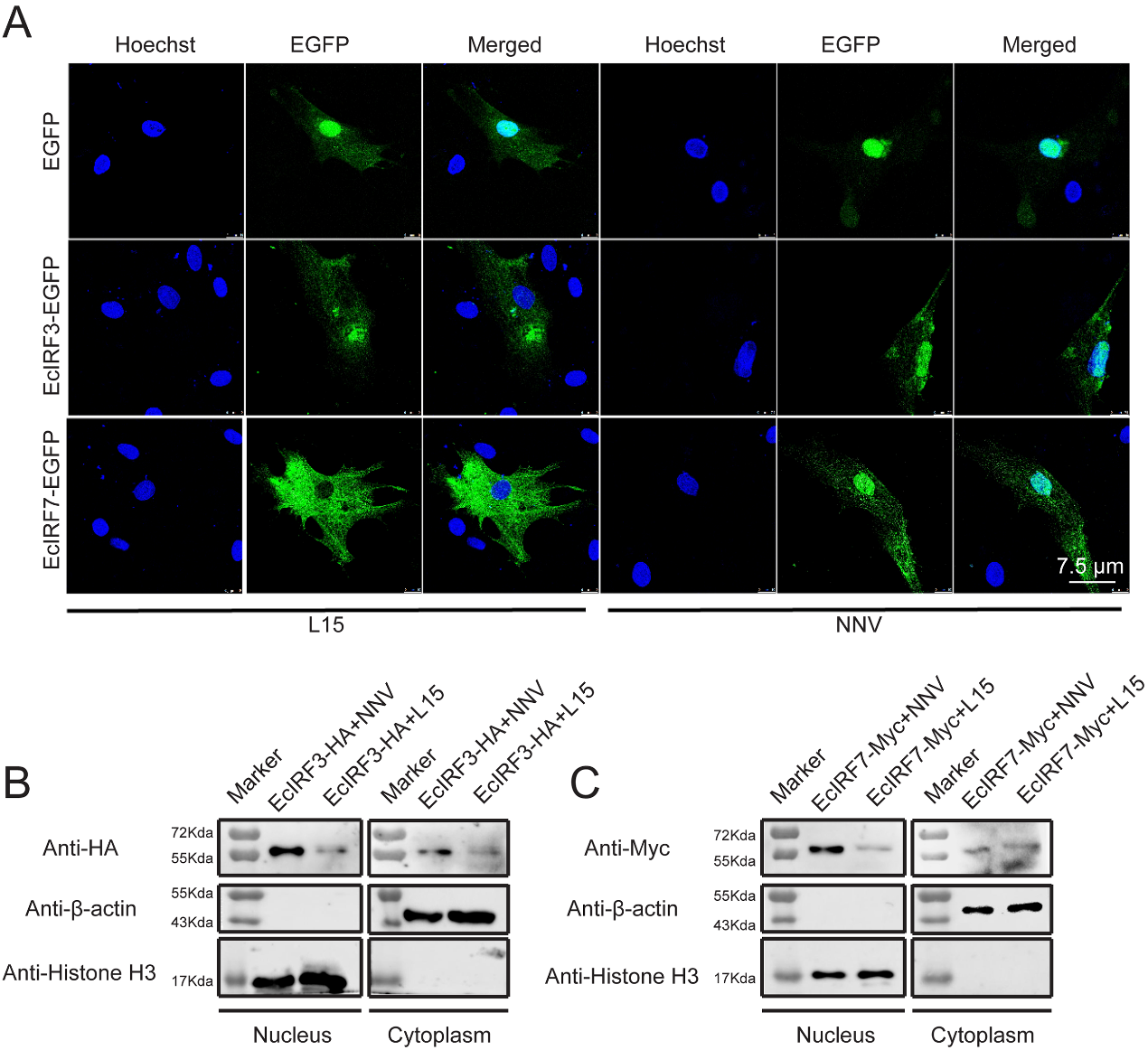


Fig. S2 OGNNV infection induced the nuclear translocation of EcIRF3 and EcIRF7 in GB cells. (A) The subcellular localization of the nuclear translocation of EcIRF3 and EcIRF7 after OGNNV infection. GB cells were transfected with EcIRF3-EGFP or EcIRF7-EGFP (Green) for 24 h, and then cells were infected OGNNV for 48 h. All nuclei were stained with Hoechst. Samples were observed under fluorescence microscopy. (B) Increasing nuclear translocation of EcIRF3-HA or EcIRF7-HA was determined by Western blotting in OGNNV-infected GB cells after 48 h of EcIRF3-HA, EcIRF7-HA or EV (HA) were transfected. Samples of nuclear portion, and cytoplasmic portion were prepared. β-actin was used as a cytoplasmic endogenous reference and Histone H3 was used as a nuclear endogenous reference for protein level standardization.


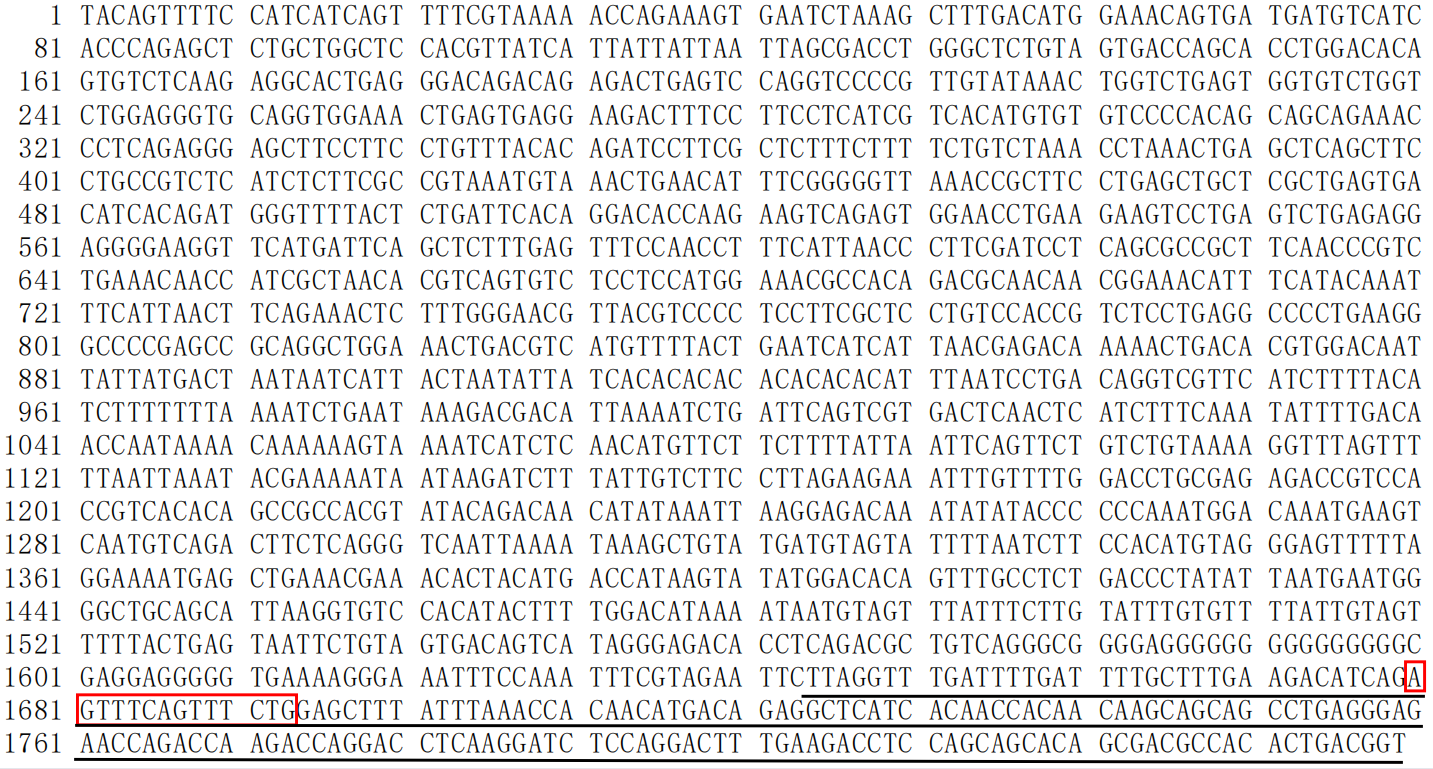


Fig. S3 The promoter sequence of EcIFNc. The red box indicates possible IRF potential binding sites. The underline indicates the 5' UTR of the EcIFNc.

Supplemental Table S1 Sequences of primer used in this paper

| Name | Sequence（5'-3'） | | Comments | |  |
| --- | --- | --- | --- | --- | --- |
| EccGAS-ORF-F | CCG**CTCGAG**GCCACCATGACTGGTAGAGGGAGACCAC | | Overexpression | |  |
| EccGAS-ORF-R  NTD-F  NTD-R  NTase+Mab21-F  NTase+Mab21-R  NTD+NTase-F  NTD+NTase-R  Mab21-F  Mab21-R  EcSTING-ORF-F  EcSTING-ORF-R  EcIRF3-ORF-F  EcIRF3-ORF-R  EcIRF7-ORF-F  EcIRF7-ORF-R  EcRNF114-ORF-F  EcRNF114-ORF-R  EcUBE3C-ORF-F  EcUBE3C-ORF-R  Q-CP-F  Q-CP-R  Q-RdRp-RT-F  Q-RdRp-RT-R  Q-EccSTING-F  Q-EccSTING-R  Q-EccGAS-F  Q-EccGAS-R  Q-EcIRF3-F  Q-EcIRF3-R  Q-EcIRF7-F  Q-EcIRF7-R  Q-EcIFNc-F  Q-EcIFNc-R  Q-EcIFNd-F  Q-EcIFNd-R  Q-EcIFNγ1-F  Q-EcIFNγ1-R  Q-EcIFNγ2-F  Q-EcIFNγ2-R  Q-EcIFIT1-F  Q-EcIFIT1-R  Q-EcISG15-F  Q-EcISG15-R  Q-EcMx1-F  Q-EcMx1-R  Q-EcTNFα-F  Q-EcTNFα-R  Q-EcIL-1β-F  Q-EcIL-1β-R  Q-EcIL-6-F  Q-EcIL-6-R  Q-EcIL-8-F  Q-EcIL-8-R  Q-β-Actin-F  Q-β-Actin-R  pGL3-EcIFNc-F  pGL3-EcIFNc-R  pGL3-EcIFNc-mut-F  pGL3-EcIFNc-mut-R  siEccGAS-1  siEccGAS-2  siEccGAS-3 | | GG**GGTACC**CTTGAAAATAGGAAAGCCCCCGTCA  CCG**CTCGAG**GCCACCATGACTGGTAGAGGGAGACCAC  GG**GGTACC**ACACCTGCCTGCCTTCTTG  CCG**CTCGAG**GCCACCATGAAAGACAAAGCTTCAGTGGACTCCA  GG**GGTACC**CTTGAAAATAGGAAAGCCCCCGTCA  CCG**CTCGAG**GCCACCATGACTGGTAGAGGGAGACCAC  GG**GGTACC**AACATGAGAGAAAGAAACCCGCC  CCG**CTCGAG**GCCACCATGAGTTACTATGAAAATCTGAAGATTTC  GG**GGTACC**CTTGAAAATAGGAAAGCCCCCGTCA  CCG**GAATTC**GCCACCATGAGAACAGAGGACAAAGAAAATC  CGC**GGATCC**TATTCTTCCTTGATAATGGTCGG  CCC**AAGCTT**GCCACCATGTCTCATTCTAAACCACTGCTC  GG**GGTACC**GTACATCTCCATCATCTCCTCGA  CCC**AAGCTT**GCCACCATGCAAAGCCCTCCAAAG  GG**GGTACC**GATCTGAGGTAGAAAAGAGTCAGC  CCC**AAGCTT**GCCACCATGGCGATGCTCGGAGGG  CGG**GGTACC**GTTGTCCAAAAGGGAGCGCTG  CCG**CTCGAG**GCCACCATGTTTAGCTTTGAGGGAGATTTCAA  CGG**GGTACC**GCTCAGCTCGAACCCGGC  ATGGTGGGAAAGCAGAACAGT  ACAGGAGTATCAGCCGACCAG  GTGTCCGGAGAGGTTAAGGATG  CTTGAATTGATCAACGGTGAACA  GTTTCGTGCCACCCATCAG  GGGGAGGTTGTCATAGAAAGTG  GGAATACAAGCGAATGCCG  GAGAAAGAAACCCGCCAAATA  GACAACAAGAACGACCCTGCTAA  GGGAGTCCGCTTGAAGATAGACA  CAACACCGGATACAACCAAG  GTTCTCAACTGCTACATAGGG  ATGCCGACCTGTAAACTGGAG  TTATTGGTTGTGACCGCAGG  CCTGCTTGAGGGGGGTTAC  GACAGCCTGCCTGCTTACAAC  CGATTCGGTCATCAAGAGCAT  CTCCGTCACGACCGACACCA  CAGCAATGGTGAGGTGGCA  TTTGCTCTGGATGATAGGGTC  ATTTGGCAGAGGAGGCT  CTTTGCTTTGGGCGACT  CCTATGACATCAAAGCTGACGAGAC  GTGCTGTTGGCAGTGACGTTGTAGT  CGAAAGTACCGTGGACGAGAA  TGTTTGATCTGCTCCTTGACCAT  GTGTCCTGCTGTTTGCTTGGTA  CAGTGTCCGACTTGATTAGTGCTT  AACCTCATCATCGCCACACA  AGTTGCCTCACAACCGAACAC  GGTTGGTCCAAGGTGTGCTTA  CTGGGATTGTCGAGGTCCTT  GCCGTCAGTGAAGGGAGTCTAG  ATCGCAGTGGGAGTTTGCA  TACGAGCTGCCTGACGGACA  GGCTGTGATCTCCTTCTGCA  CGG**GGTACC**TACAGTTTTCCATCATCAGTTTTCG  CCG**CTCGAG**ACCGTCAGTGTGGCGT  TTTGATTTTGCTTTGAAGACATCAGGAGCTTTATTTAAAC  TCATGTTGTGGTTTAAATAAAGCTCCTGATGTCTTCAAAG  GCAGAAGTCATCAATAGAA  CAGGAACGAAGTGAAGAAA  CAAGCGAATGCCGTATTAT | | Overexpression  Overexpression  Overexpression  Overexpression  Overexpression  Overexpression  Overexpression  Overexpression  Overexpression  qPCR  qPCR  qPCR  qPCR  qPCR  qPCR  qPCR  qPCR  qPCR  qPCR  qPCR  qPCR  qPCR  qPCR  qPCR  qPCR  qPCR  qPCR  Dual-luciferase  Dual-luciferase  siRNA | |

Nucleotides in bold represent the restriction sites introduced for cloning.
